# The effects of the form of sugar (solid vs. beverage) on body weight and fMRI activation: A randomized controlled pilot study

**DOI:** 10.1371/journal.pone.0251700

**Published:** 2021-05-17

**Authors:** John W. Apolzan, Owen T. Carmichael, Krystal M. Kirby, Sreekrishna R. Ramakrishnapillai, Robbie A. Beyl, Corby K. Martin

**Affiliations:** 1 Pennington Biomedical Research Center, Louisiana State University System, Baton Rouge, LA, United States of America; 2 Department of Physics and Astronomy, Louisiana State University, Baton Rouge, LA, United States of America; Università degli Studi di Milano, ITALY

## Abstract

**Objective:**

To test if sugar sweetened beverages (SSBs) and sugar sweetened solids (SSSs) have differential effects on body weight and reward processing in the brain.

**Methods:**

In a single blind randomized controlled pilot trial (RCT), twenty participants with BMI between 20 and 40 kg/m^2^ were randomized to consume a 20 fluid ounce soda (SSB, 248 kcal) or the equivalent in solid form (SSS; similar to thick gelatin or gummy candy) daily. At baseline and day 28, fasting body weight and fed-state BOLD fMRI of the brain were assessed. Differences in fMRI signals between views of low-fat (LF (<30%)) high sugar (HS (>30%)) food, and non-food images were calculated in brain regions implicated in energy homeostasis, taste, and reward.

**Results:**

All participants in the SSB (6F 4M; 8 Caucasian; 36±14 y, 28.2±5.5 kg/m^2^; Mean±SD) and SSS (3F 7M; 6 Caucasian; 39±12; 26.3±4.4) groups completed the study. Weight change was 0.27±0.78 kg between SSB and SSS participants. Changes in the fMRI response to LF/HS foods in reward, homeostatic and taste regions tended to not be different between the groups over the four weeks. However, activation of the right substantia nigra increased following the SSB but decreased activation following the SSS in response to LF/HS foods over 28 days (-0.32±0.12). Ratings of wanting for LF/HS foods were correlated with activation in several brain regions, including the OFC.

**Conclusions:**

Change in weight was modest between the groups in this study. Daily consumption of a SSB over 28 days led to mixed responses to LF/HS foods in areas of the brain associated with reward. Ratings of wanting are correlated with fMRI activation inside an MRI scanner.

## Introduction

Food form refers to the physical state of foods, i.e., if food is in beverage (liquid) or solid form. Compared to solid foods, adults do not fully compensate for calories consumed in beverages in short-term preload studies [1–4], suggesting that food form may have an independent effect on energy intake and body weight [4–6]. However, short-term intake studies may fail to accurately capture compensatory behavior over the longer term. Previously it has been shown that in humans, compensatory changes in food intake typically occur over 2–6 days [7, 8], which questions the validity of preload studies. To our knowledge, only one study to date has investigated the effect of food form ingested as carbohydrate on energy intake and body weight, though the foods differed slightly on other parameters than liquid vs. solid [5]. That study found that over four weeks, there was no difference in body weight change between carbohydrate beverage and isoenergetic solid food form [5]. In another four-week study, participants who consumed sugar-sweetened beverages (SSBs) gained far less weight than expected should no compensation have occurred [9]. It is clear from the small number of studies in this area that the effects of food form on energy intake and weight gain are underexplored, particularly when the products are identical except for their form. Addressing this question is important given the strong interest in additional research on the extent to which compensation occurs over the long-term in response to energy from SSBs [10].

In addition to a relative lack of data on body weight, there is a paucity of data on the effects ingesting foods of differing forms, particularly carbohydrate, may have on brain responses to visual food cues. Responses to visual food cues in reward- and motivation-processing regions of the brain are elevated in individuals with obesity compared to normal weight individuals [11] and also fail to decrease in individuals with obesity after feeding [12]. Additionally, prolonged exposure to highly palatable energy-dense food has been shown to reduce brain responses to visual food cues in reward-related regions [13, 14]. Initial research also suggests that greater reward activation responses to visual food cues may predict weight gain and success of weight loss [15]. Repeated exposure (over 3 weeks) to SSBs decreases response to frontostriatal brain regions suggesting alterations in reward pathways [16] which could lead to hyperphagia, but whether this response is specific to SSBs, as opposed to solid sugar, is under-explored.

Lastly, there is little data on the effects food form may have on cravings and preferences for food items. Our previous work utilizing self-report scales has found that repeatedly consuming certain types of foods conditions the body to prefer and crave those foods, while restricting intake of foods conversely reduces cravings for the types of foods that are restricted [17–22]. This is consistent with Burger [16] who found that consuming a specific, novel SSB for 3 weeks resulted in decreased pleasantness ratings and desire to consume other beverages that were not consumed. Further, people have higher reward sensitivity for foods that are more frequently consumed and, interestingly, this effect is specific to less healthy foods, including sweet snacks and SSBs [23].

The effect of long-term consumption of SSBs or Sugar Sweetened Solids (SSSs) on body weight and fMRI activation are not well understood. This study allowed us to engineer our products and evaluate acceptability ratings for the SSB and SSS. The purpose of the current pilot study was to address these knowledge gaps by pilot testing if weight, brain responses to food cues in the fed state, and food cravings and preferences over 4 weeks of consumption of a specific food item changed differentially in groups that consumed the same food in different form.

## Materials and methods

### Ethics

The study reported herein was conducted according to the guidelines in the Declaration of Helsinki. All participants were given verbal and written explanations about the study, provided written informed consent, and received a monetary stipend. The study was approved by the Pennington Biomedical Institutional Review Board (IRB; FWA 00006218). The study was registered at ClinicalTrials.gov (NCT 03190993). Since it was a pilot study, only the primary outcome of body weight was reported in the NCT registry. After the study was initiated, BMI was expanded from 35 kg/m^2^ to 40 kg/m^2^.

### Participants

Men and women without diabetes aged 21 to 65 years with a BMI range of 20 to 40 kg/m^2^ were recruited. All inclusion/exclusion criteria were based upon self-reported medical history/checklist except for body mass index (BMI). Other key inclusion criteria included participants being willing to consume study foods and willingness to archive their neuroimaging data. Participants were asked to maintain their regular SSB consumption (> 7, 12 oz. sugar-sweetened beverages per week) in addition to consuming the foods provided during the study (i.e., either a daily SSB or SSS). Exclusion criteria included prediabetes, Type I or II diabetes, smoking (former smokers must be smoke free for 6 months), cancer not in remission, serious digestive disorders, uncontrolled thyroid disorder, non-weight stable, conditions that affect metabolism or body weight, intentions of becoming pregnant or current pregnancy, uncorrected vision problems, color blindness, left handedness, current or past alcohol or drug abuse (> 3 drinks/day of any alcoholic beverage), or contraindications to magnetic resonance imaging (MRI).

Recruitment at Pennington Biomedical Research Center (PBRC) was centralized. PBRC communications and marketing specialists created advertisements for our studies and media coverage to boost recruitment efforts. Our recruitment strategies used targeted advertisements on social media (Craigslist as well as paid ads on Facebook, Instagram, and Twitter) and e-mail blasts through our e-mail listserv. We also used traditional methods of face-to-face recruiting and flyer drops that target community venues including branches of the local public library, commercial retail outlets (YMCA, restaurants, etc.), and community parks and recreational facilities.

### Study design

**Fig 1:**
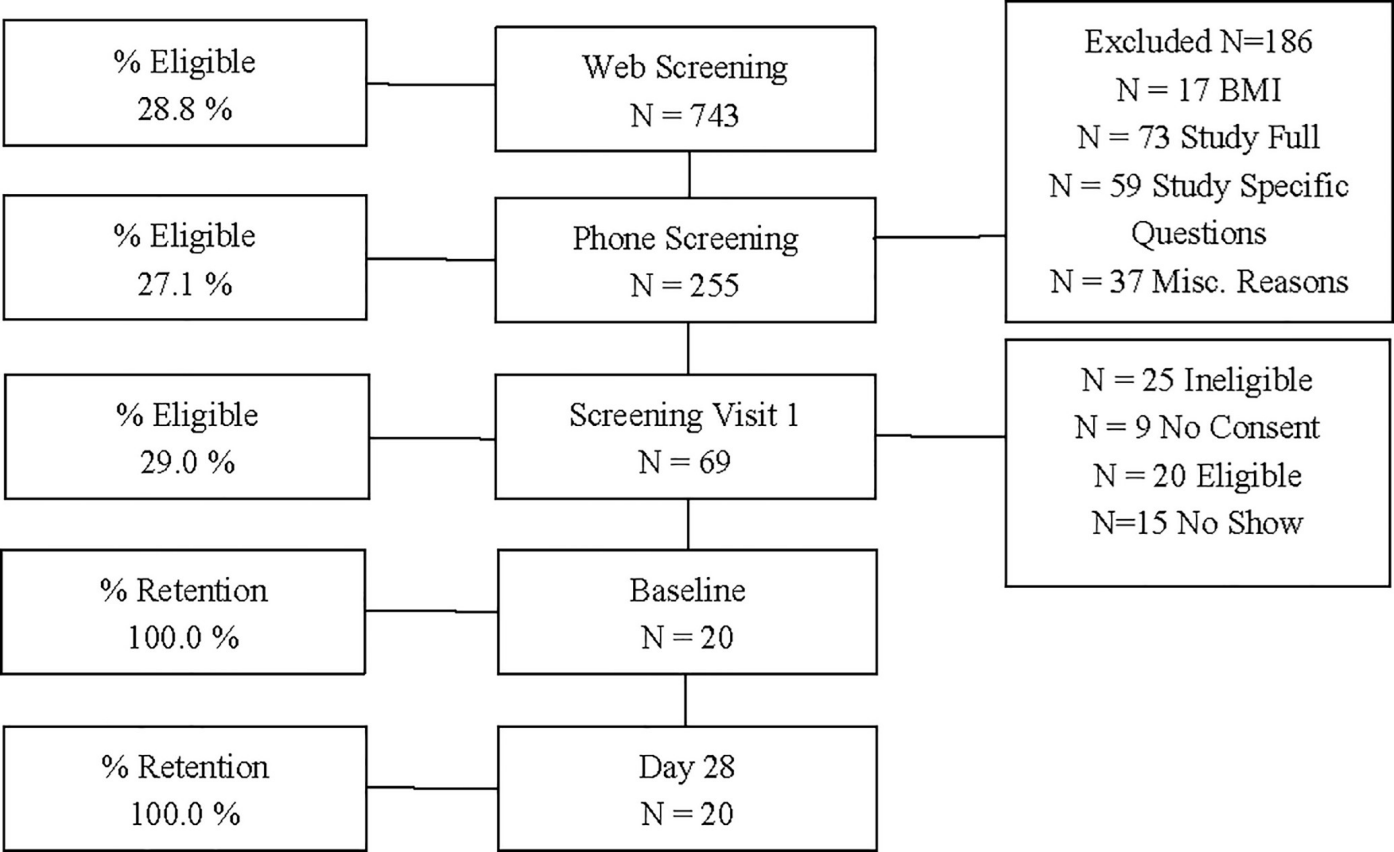
CONSORT diagram.

This pilot study was a single-blinded parallel arm randomized controlled trial examining SSB and a nutritionally equivalent SSS with a 1:1 allocation ratio performed at Pennington Biomedical Research Center in Baton Rouge, Louisiana from July 2017 till June 2018. Stratified block randomization was performed by the biostatistician using SAS v9.4 within each BMI group (normal weight, overweight, or obese) with randomized blocks of size 2. Investigators and clinic staff obtaining outcome assessments (i.e. body weight) were blinded to participant treatment. Due to the nature of the study, participants could not be blinded. Thus participants and intervention staff providing the product and checking compliance were not blinded. The Consort Diagram is shown in Fig 1. Participants consumed products daily for 28 days with assessments occurring at baseline and day 28. During a screening visit, participants were consented and a fasting weight and height were obtained. Also participants completed a taste test to ensure that they would be willing to consume the study foods. The taste test relied on a 9-point Likert scale, anchored from 1 (Dislike Extremely) to 9 (Like Extremely), and asked participants to rate the texture, flavor, and overall acceptability of both products, as well as the ability to eat the product for 28 days (answered as ‘yes’ or ‘no’). Then, individuals were excluded if they stated they were unable to eat the product for 28 days.

At the study assessment visits, participants arrived at the biomedical research center well rested following a 10–12 hour fast. Fasting body weight was obtained by a trained clinic coordinator. A series of psychological assessments were performed including:

The Retrospective VAS (RVAS) is used to measure subjective ratings of hunger, satiety, desire to eat, fullness, and prospective food consumption over the past week. When completing RVAS, participants rate the intensity of these subjective states on a line anchored from “not at all” to “extremely.” The line is divided into 100 equal units. Research supports the reliability and validity of RVAS for measuring subjective states related to food intake [24].

The Food Craving Inventory (FCI) is a 33-item measure of specific food craving. The FCI assesses the frequency with which an individual experiences a craving for a particular food. The measure consists of 5 empirically-derived factors: High fats, Sweets, Carbohydrates/starches, Fruits/Vegetables, and Fast food fats. All items are scored in a 1 (Never) to 5 (Always) multiple choice format. The measure has demonstrated good reliability and validity [25, 26].

The Barratt Impulsiveness Scale (BIS) is a 30-item self-report measure describing impulsive or non-impulsive behaviors and preferences. The scale consists of 6 first order factors: Attention, Cognitive Stability, Motor, Perseverance, Self-Control, and Cognitive Complexity; and 3 second order factors: Attentional, Motor and Non Planning. The items are rated on a 4-point scale with responses ranging from 1 (Rarely/Never) to 4 (Almost Always /Always). The measure assesses the personality/behavioral construct of impulsiveness [27], and has demonstrated good psychometric properties in multiple populations [28].

The Yale Food Addiction Scale (YFAS) [29] is a 27-item self-report questionnaire used to identify individuals showing tendencies for addictive-like behaviors towards certain types of foods, such as those high in fat or sugar. The measure also allows subjects to subjectively identify specific problem foods.

Urine pregnancy test was performed on females (and read by medical investigator prior to MRI). Lastly, functional MRI (fMRI) was performed.

The baseline clinic visit occurred approximately within one month after the screening visit. For females, the baseline visit, and the follow-up visit 28 days later, occurred during the luteal phase of the menstrual cycle. Participants were stratified based on BMI class and treatment. Each group had at least 3 participants with normal weight, overweight, and obesity based on BMI. Participants returned to the center weekly for adherence checks and provision of study foods.

### Treatments

**Fig 2:**
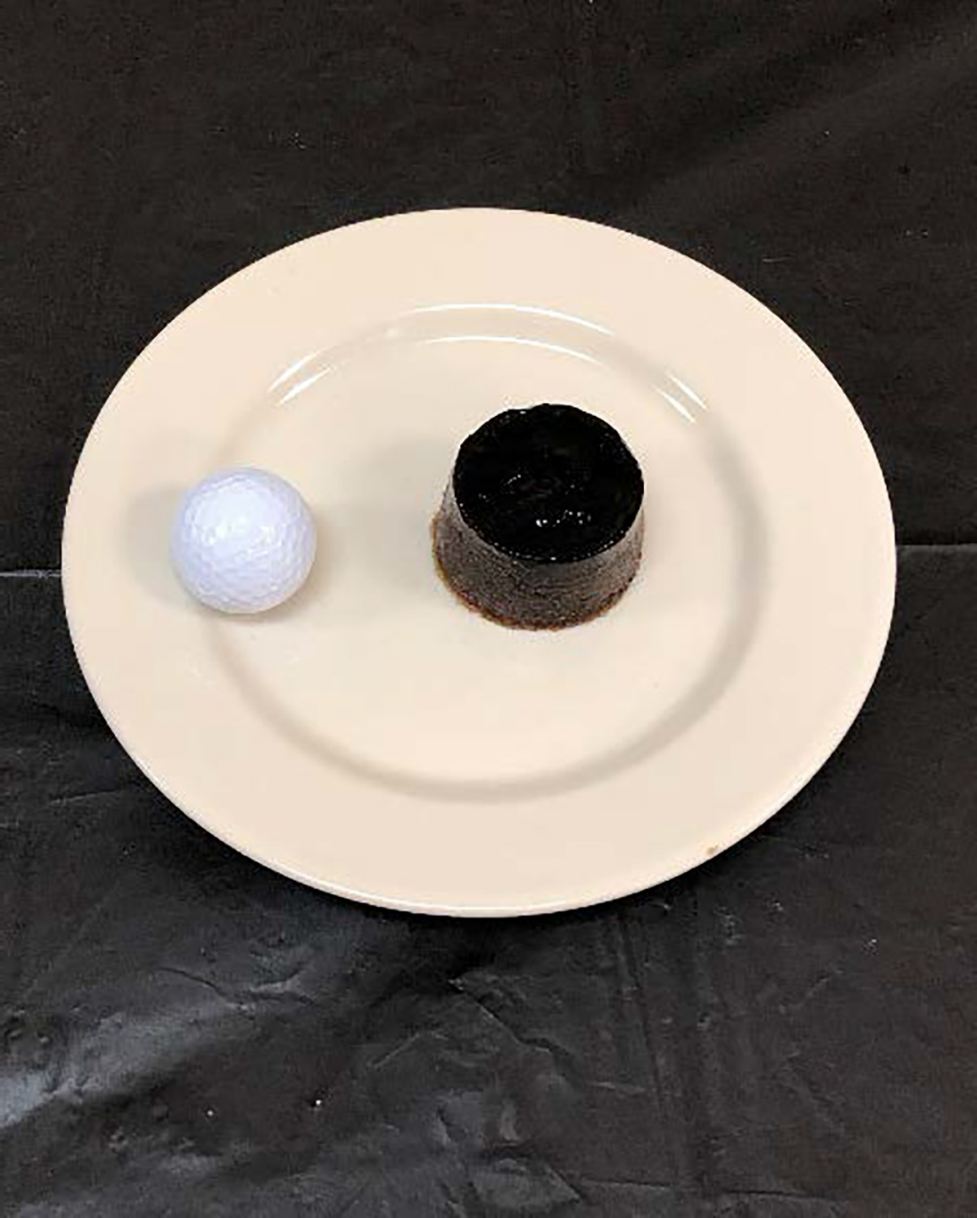
The Sugar Sweetened Solid (SSS). The golf ball provides scale.

The SSB was a 20 fluid oz. Coca-Cola® without a label. It was augmented with 2.1 g of whey protein powder (ProCel, Global Health Products, Inc., Rochester, NY) to match the additives in the gelatin (Knox; E.D.Smith® Foods, Ltd.) that was used to make the SSS. The SSB was 248 kcal. The SSS was made with Coca-Cola® syrup concentrate (i.e. fountain syrup) and provided equal energy content to the 20 oz. SSB (248 kcal). It included 3.0 g of gelatin to produce the solid form (Fig 2). It also included 3.5 mg sodium from NaCl, 11.1 mg potassium from KCl, and 11.5 mg calcium from CaCl (all from Letco Medical, Wayne, PA), which were included to make it nearly identical to the SSB (energy, macronutrients, and micronutrients). Thus, the difference between products was the fluid (i.e. water content) between the SSB and SSS.

### Intervention

Consumption of the product associated with participants’ random assignment began during the baseline assessment. At this time, participants consumed their randomized group assignment (either a SSB or SSS) prior to their fMRI scan. At the end of this study visit, 7 days’ worth of products were provided. A few rescue products were also provided in case they could not return to the center in 7 days to receive additional product. The SSS rescue products were provided frozen. Participants were instructed to consume their randomized product daily, but each day it could be consumed *ad libitum* (e.g. timing of product consumption was chosen by participant). Participants came to the center weekly thereafter (approximately day 7, 14, and 21) for product pick-up as well as an adherence check with the study interventionist. Daily adherence was quantified over the prior week based on multiple pass questioning of the participant and counting empty bottles and containers.

### Functional Magnetic Resonance Imaging (fMRI)

MRI scanning was completed on a 3T General Electric Discovery 750W with 32-channel head coil. Blood-oxygen-level-dependent (BOLD) fMRI acquisition parameters include repetition time of 3000ms, echo time of 30 ms, flip angle of 90, slice thickness of 3.5mm, and 64x64 image matrix. As in prior work [30], a T1-weighted magnetization prepared rapid gradient echo (MPRAGE) was collected as an anatomical reference for functional data.

fMRI was used to assess brain responses to food and non-food control images. Scans were conducted 30 min after ingesting the participants randomized food product (i.e., either the SSB or SSS) at baseline and day 28, and food product ingestion followed an overnight fast. The fMRI food image task was developed using images from the Food-pics database [31], to which we applied a previously described macronutrient categorization [32]. Foods were classified as low fat (LF, <30%) and high sugar (HS, >30%). This resulted in 15 images in the food category. Fifteen (15) non-food control images of everyday household objects were also shown. Each 600 × 450 color photo showed one food on a white background.

Images were shown in an event-related design. In each trial of the fMRI task, one food image was displayed for 5s followed by 0.5s of a fixation crosshair. The foods in the LF/HS category were divided into 1) fruits and vegetables: apple, watermelon, green pepper, grapes, banana, carrot, green beans, orange, pickle, tomato; and 2) sweets: lollipop, gummies, cola gummies, gummy bears, chocolate marshmallows, and gumdrops. Next, the same image scaled to 80% of the original size was shown with the words “How much do you want to eat this?” for 2.5 s. A slider bar with “Not at all” and “Want very much” on the left and right ends appeared. The participant moved a joystick to select a slider bar position and clicked a joystick button to finalize the response. The response was recorded as the wanting rating for that image. A fixation crosshair displayed for a minimum of 1.5 s before the next trial. The task consisted of consecutive trials, with all images shown in a counterbalanced order that was the same across participants.

### fMRI activation analysis

Responses to the 15 food images in the LF/HS category (to match the macronutrient profile of the foods consumed herein) along with the 15 non-food control images were included in the analysis. Preprocessing of fMRI data in Statistical Parametric Mapping (SPM) version 8 included slice timing correction, head motion correction, smoothing, and warping to a standard coordinate frame. The sources of artifacts in time series data including spiking and motion were identified and removed during the first level analysis. Time points (image volumes) with head rotation greater than 2° or translation greater than 2 mm or z-normalized global brain activation (standard deviations away from the mean) above 2.3 were considered as outliers. These bad time points were omitted in the General Linear Model (GLM) by including a single regressor for each outlier volume. Artifact Detection Toolbox (ART) was used to identify outliers and create regressors. Data were entered into a first-level voxel-wise analysis with each trial modeled as a boxcar function that covers the period of time when the large image is viewed. The boxcar function was convolved with the canonical hemodynamic response function. The BOLD signal contrast between LF/HS and control conditions (hereafter, “fMRI activation”) was calculated at a set of 3D ROI coordinates taken from a recent meta-analysis [33]. The fMRI activations at all ROIs located inside a specific hemisphere of an anatomical area (such as the right hypothalamus) were averaged together to create two estimates for that anatomical area, right and left. The ROIs were in anatomical areas implicated in energy homeostasis (hypothalamus), reward and motivation (amygdala, dorsal striatum, ventral striatum, inferior orbitofrontal cortex or OFC, lateral OFC, prefrontal cortex, putamen, substantia nigra, caudate, cingulate gyrus, medial OFC, and insula). Coordinates are shown in S1 Table. Secondarily, regions associated with taste (thalamus, postcentral gyrus, frontal operculum / anterior insula, operculum) were examined. Since a set of a priori defined brain regions were tested in this pilot study, fMRI analyses were exploratory and no multiple comparisons adjustment was performed.

### Data and statistical analysis

Parts of these study data were collected and managed using REDCap electronic data capture tools hosted at Pennington Biomedical Research Center. REDCap (Research Electronic Data Capture) is a secure, web-based application designed to support data capture for research studies, providing: 1) an intuitive interface for validated data entry; 2) audit trails for tracking data manipulation and export procedures; 3) automated export procedures for seamless data downloads to common statistical packages; and 4) procedures for importing data from external sources [34].

Sample size was based on previous published literature suggesting at least 10 participants a group and about 10% of future sample is sufficient for pilot studies [35]. Analyses were carried out using SAS, Version 9.4 (SAS Institute, Cary, NC). Linear models modeling change (Day 28 –Baseline) were used to test if treatment groups differed. Normality of the residuals from the mixed model were checked and observations with a residual value of ± 3 were investigated. Similar models were used with fMRI activation. Pearson correlation between average wanting ratings for fruits/vegetables and sweets versus energy homeostasis, reward and motivation, and taste fMRI activation contrasts were calculated. Data are reported as mean ± SD and/or 95% confidence intervals.

## Results

### Participants

Participant characteristics are shown in Table 1. Adherence during the 28 day study was similar between groups (SSB, 99.3 ± 1.5%; SSS, 100.4 ± 3.3%) and retention was 100%. Taste test ratings of texture, flavor, and acceptability were similar between SSB and SSS.

**Table 1:** Participant characteristics.

	SSB	SSS
Sex	3 F, 7 M	6 F, 4 M
Race	6 White, 3 Black, 1 Asian	8 White, 1 Black, 1 Asian
Age (y)	36 ± 14	39 ± 12
BMI (kg/m^2^)	28.2 ± 5.5	26.3 ± 4.4
Weight (kg)	79.0 ± 16.9	81.4 ± 15.9
Taste Test		
Texture	7.6 ± 0.8	7.0 ± 1.3
Flavor	7.4 ± 1.1	6.6 ± 01.5
Acceptability	7.5 + 0.8	7.0 + 01.2

Mean ± SD. SSB, Sugar Sweetened Beverage; SSS, Sugar Sweetened Solid. No significant differences were detected between groups.

### Anthropometrics

Change in body weight over 28 days was 0.27 ± 0.78 kg [-0.49, 1.03] between the SSB and SSS conditions. This was driven by a weight increase in the SSB group (0.38 ± 0.94 kg; -0.29, 1.05) and very smaller increase in the SSS group (0.11 ± 0.56 kg; -0.31, 0.54).

### Self-report questionnaires

Changes in ratings of perceived appetite on the RVAS did not suggest differences between the two groups over time (S2 Table). The fruits/vegetables subscore slightly increased in the SSB group and slightly decreased in the SSS group for a between group change of 0.5 ± 0.4. High fats, carbohydrates, and the total score increased in the beverage group and the solid group resulting in group change differences of -0.2 ± 0.3, 0.1 ± 0.3, and 0.1 ± 0.2, respectively. With the BIS, cognitive complexity increased in the SSB group but decreased in the SSS group resulting in a difference of 1.1 ± 1.1. In the motor 1^st^ order factors, SSB and SSS were increased at the 28 day follow-up time point, with SSB and SSS increasing its score leading to a between group change of -2.0 ± 2.2. No other impulsiveness factors suggested differences between groups. YFAS did not differ between groups over time.

### fMRI

Changes in fMRI activation over the four weeks of treatment within energy homeostasis, reward, and taste areas are reported in the S3 Table.

With reward, the insula and substantia nigra in the SSB group increased activation and the SSS group decreased activation over 28 days leading to a changes of 0.83 ± 0.7 and 0.39 ± 0.31, respectively. With energy homeostasis, the hypothalamus decreased activation in the SSB group but increased activation in the SSS group leading to a change of -0.4 ± 0.43.

### Wanting

At baseline, associations between average wanting ratings for LF/HS foods and fMRI activation to the same images were seen. In the right hemisphere, fMRI activation for LF/HS foods in the cingulate gyrus was associated with wanting of fruits and vegetables (ρ = 0.51). In the left hemisphere, fMRI activation for LF/HS foods in the insula and medial OFC were positively associated with wanting for sweets (ρ = 0.33; ρ = 0.40, respectively), whereas the fMRI activation for LF/HS foods in the hypothalamus was positively associated with wanting for fruits and vegetables (ρ = 0.33).

The association between the change in average wanting for LF/HS foods and change in fMRI activation over 28 days was limited. In the right hemisphere, change in fMRI activation for LF/HS foods in the inferior OFC was negatively correlated with change in wanting for sweets (ρ = -0.37). In the left hemisphere, change in fMRI activation for LF/HS foods in the lateral OFC was negatively correlated with change in wanting for fruits and vegetables (ρ = -0.31). No other medium effect sizes were seen.

## Discussion

In this study, individuals were randomized to consume nutrient-identical SSS or SSB products daily for 28 days. Participants exhibited a modest change in body weight between groups. Also, the consumption of added sugar in beverage or solid form led to limited differential group changes in brain activation to visual food cues. As shown in the S3 Table, some of the fMRI activation findings had large variability, including at baseline, thus the potential for significance was greatly reduced. However, the majority of reward regions demonstrated decreased (blunted) reward suggesting this is in line with previous data responses to SSBs [16]. Nevertheless, our data were somewhat mixed. With SSB consumption fMRI activation in some reward regions tended to increase, and fMRI activation in response to LF/HS foods in the substantia nigra tended to result in between group differences. Overall, the responsiveness to LF/HS images tended to demonstrate some changes in fMRI activation over 28 days of SSB vs. SSS consumption. Lastly, wanting ratings given within the scanner were associated with simultaneous fMRI activation in homeostatic and reward regions of the brain. To the authors’ knowledge, this is the first time this effect has been shown.

The mechanism for the potential differential response to food intake and body weight is not definitively known. However there are a variety of theories including that human adults do not to fully compensate for energy consumed in beverages, which leads to a positive energy balance and weight gain, is largely supported by short-term preload studies [1–4, 36]. The lack of dietary compensation could be due to cognitive factors [37] (also including fMRI activation), GI transit time and gut hormones [37–39], differences in intake (i.e. snacking behaviors) [40], and requires further study. Some longer-term previous studies examined the effects of beverages vs. solid food on body weight in animals [41, 42] as well as humans. The human work included examining the effects of food form on consumption of fruit and vegetables [43], meal replacement products [44], and sugars [5]. The work on sugar also spanned 28 days and demonstrated similar effects to our own. In that study [5], jelly beans and caffeine free soda were provided so the products were similar but not identical like our products. The study foods were isocaloric and 450 kcal was provided daily. The study was a within-subjects crossover design in 15 younger participants who on average had a normal BMI. There was no difference between groups with the solid group gaining 0.3 kg, the beverage group gaining 0.5 kg. Thus a 0.2 kg difference was present between the groups [5]. This suggested that the beverage may lead to greater weight gain than the solid but the results were modest. We utilized identical products and a stratified study design that recruited a similar number of persons whom were normal weight, overweight, and obese. Interestingly the results were similar with a 0.27 kg difference between groups found in the current study. Based on the current and previous studies it is unclear if beverages lead to greater weight gain than solid foods, particularly foods high in added sugar, over the long-term. A well-powered follow-up study powered based on these pilot data is needed to examine if there are differential effects of the consumption of various forms of added sugar on body weight.

The SSBs (and SSS) incorporate both sugars (glucose and fructose) since high fructose corn syrup is utilized in the food products. Ingestion of glucose and fructose previously caused differences in regional cerebral blood flow with glucose, but not fructose, activating brain regions shown to regulate reward and appetite [45]. Thus, we speculate that glucose may be playing a stronger role in causing these differential changes in brain reward compared to fructose.

Despite body weight being similar over 28 days, we saw trends for differences in brain activation in areas of the brain associated with reward in the SSB group. Brain activation to LF/HS foods predominantly decreased following the ingestion of the SSB for 28 days. This finding is similar to previous studies showing reduced reward response to a milkshake following frequent consumption of ice cream [13]. However, the SSS, which only differed from SSB in the water content and the way it was ingested, resulted in limited changes to brain activation to the LF/HS images. Specifically, the addition of the fat content along with the carbohydrate in previous studies [13, 16] may have led to the slightly differential responses compared to the current study. The substantia nigra, right insula, and hypothalamus may be responsive to either the form of energy ingested (i.e. beverage vs. solid) or the macronutrient content. The LF/HS food images included foods that are similar to the SSS (i.e. lollipops, gummy bears, gum drops) which may have played a role in this response. This is important as it identifies possible brain mechanisms that explain why chronic consumption of certain food products, such as SSSs or SSBs, affects preference and possibly selection and intake of specific food groups. This suggests that the form of food may influences activation in areas of the brain which have been associated with eating behavior and body weight.

It is critical to better understand if beverages lead to differential effects on body weight and health compared to solid foods. Public health policies are specifically targeting SSB intake [46–49], but not solid foods that are high in added sugar (e.g., candy, cakes, cookies, pies, pastries). This suggests that regulation of SSBs rather than sugar in all forms may be viewed as an especially effective means to prevent weight gain and poor cardiometabolic health [50, 51]. However, there is little to no definitive evidence indicating that added sugar from SSBs is more harmful than added sugar from solid foods. This knowledge gap is particularly concerning given the focus on taxing SSBs. For example, the Society for Behavioral Medicine has chosen to recommend an excise tax of at least 20% specifically on SSBs [49]. Yet, more evidence is needed [52–54] to inform policy directives and decisions and perhaps the lack of empirical data in this area has led to seemingly disparate recommendations by organizations and local policy makers.

Some strengths and innovation of the pilot study included products that were created. This study utilized a stratified study design thereby incorporating persons of normal weight, overweight, and obesity. It also tested visual images that had a similar macronutrient composition as study foods and examined fMRI activation of wanting the visual images inside the scanner. This study did have some limitations. These included a small sample size and subsequent low power, and the lack of a control group. However, future studies can utilize the 95% confidence intervals to power future work. Also, the LF/HS images included foods in solid form thus were representative of the macronutrient composition but not necessarily the food form of the SSB exposure (i.e. beverages). Lastly, psychological assessments were provided but no specific exclusion criteria was provided for persons with reported eating disorders.

Following 28 days of ingesting an equivalent product that only differed in food form, no statistical differences in body weight between groups were shown. However, brain activation in response to LF/HS foods diverged over the 28 days in the SSB group within brain regions associated with reward and motivation. Understanding the neural and physiological consequences of consuming added sugar in different forms will better inform current efforts to reduce its consumption.

## Supporting information

S1 Checklist CONSORT 2010 checklist of information to include when reporting a pilot or feasibility trial*. (DOC)

S1 Table Coordinates for each region of interest. (DOCX)

S2 Table Change in appetitive sensations, food craving, and impulsiveness in adults consuming isocaloric beverages and solids for 28 days. (DOCX)

S3 Table Change in fMRI activation in adults consuming isocaloric beverages and solids for 28 days. (DOCX)

S1 Protocol The Diet composition and Energy Balance Pilot Study (The DEB Pilot Study). (PDF)

S1 Dataset (XLSX)
