# Supplementary material for: The effects of the form of sugar (solid vs. beverage) on body weight and fMRI activation: A randomized controlled pilot study
Source: PLoS One. 2021 May 17;16(5):e0251700. doi: 10.1371/journal.pone.0251700 (PMC8128228; doi:10.1371/journal.pone.0251700)
Supplement: S1 Table — (DOCX) [file pone.0251700.s002.docx]

S1 Table. Coordinates for Each Region of Interest

Reward MNI Coordinates

Region Hemisphere X Y Z

Amygdala R 15.9 -2.5 -24.8

Amygdala R 19.2 -11.8 -21.7

Amygdala R 20.3 -4.6 -24.7

Amygdala L -22.9 -2.4 -21.9

Amygdala L -38.1 -8.2 -25.5

Amygdala L -27.3 -8.2 -25.7

Amygdala L -19 -8 -16

Amygdala L -18 0 -14

Amygdala L -22 -2 -20

Caudate R 15 -18 21

Caudate R 7.4 7.4 -9.9

Caudate R 13.9 24.9 -7.3

Caudate R 4.5 11.7 13.2

Caudate L -7.7 25 -4.7

Caudate L/R 3.4 12.8 13.1

Cingulate Gyrus R 14.4 22.9 28.8

Cingulate Gyrus L -4.9 10.9 39.2

Cingulate Gyrus L -6 24 15

Cingulate Gyrus L -7.7 48.9 -2.6

Cingulate Gyrus l -5 26.9 37.7

Cingulate Gyrus L -2 30 0

Cingulate Gyrus L -18 30 18

Cingulate Gyrus L -4 -34 26

Cingulate Gyrus L 0 48 0

Cingulate Gyrus L -2 36 10

Dorsal Striatum L -18.6 22 -13.2

Dorsal Striatum L -8 10 -8

Dorsal Striatum R 20.4 13.9 -10.8

Lateral OFC L -30.5 47.7 -13.3

Lateral OFC L -37 35.7 -15.3

Lateral OFC L -25 31 -17

Lateral OFC R 27.9 36.2 -13.1

Medial OFC R 5.1 58.5 -14.9

Medial OFC R 3 53.9 -6.6

Medial OFC L -1.4 61.7 -15.2

Medial OFC L -8 28 -20

Inferior OFC L -36 30 -16

Inferior OFC L -36 22 -16

Inferior OFC R 22 24 -24

Inferior OFC R 38 24 -22

Insula L -35.6 17.5 7.7

Insula L 43.1 2.4 8.2

Insula L -35 14 10

Insula L -38 5 -8

Insula L -37 -5 7

Insula R 40 6 -10

Insula R 39 -6 8

Insula L -38 -10 4

Insula R 40 6 -12

PFC L -3.2 58.2 16.6

PFC L -4.5 0.7 68.3

PFC R 14.8 4.4 60.8

PFC R 10.3 32.4 50.3

Putamen R 28 -4 0

Putamen R 26 -8 14

Putamen L -12 0 -2

Substantia Nigra R 9.6 -23.4 -18.1

Substantia Nigra L -5.4 -29.2 -10.6

Ventral Striatum R 22 12 -8

Ventral Striatum R 6 0 -12

Ventral Striatum L -9 6 -6

Energy Homeostasis

Hypothalamus R 7.4 2.4 -18.4

Hypothalamus R 7.4 -10.6 -19.4

Hypothalamus R 9.5 -4 -17.8

Hypothalamus L -5.6 -8.1 -14.9

Hypothalamus L -7.7 -10.3 -14.7

Hypothalamus L -7.7 2.9 -11.5

Hypothalamus R 6 6 -6

Taste and Sensory

Frontal Operculum R 36 9 9

/ Anterior Insula

Frontal Operculum R 56 1 28

/ Anterior Insula

Frontal Operculum R 56 18 16

/ Anterior Insula

Frontal Operculum L -39 0 6

/ Anterior Insula

Insula L -45.5 -1 13.8

Insula L -33.5 19 1

Insula L -42.1 15.3 8.1

Insula L -40 16.6 -1.1

Insula R 42.1 18.8 -4.9

Insula R 46.3 16.3 -9.2

Insula R 42 18 -14

Insula R 40 -2 4

Insula R 26 24 -6

Operculum R 42 -28 22

Postcentral Gyrus R 66.2 -26.2 17.1

Postcentral Gyrus L -61.4 -7.7 15.2

Postcentral Gyrus L -39 -50 59

Thalamus L -15 -27 -3

Thalamus L -7.5 -29.5 -2.7

Thalamus R 3.1 -25.3 -15.6

PFC, Prefrontal Cortex; OFC, orbitofrontal cortex. Coordinates for each brain region hemisphere were averaged for the analyses.
