# Supplementary material for: The effects of the form of sugar (solid vs. beverage) on body weight and fMRI activation: A randomized controlled pilot study
Source: PLoS One. 2021 May 17;16(5):e0251700. doi: 10.1371/journal.pone.0251700 (PMC8128228; doi:10.1371/journal.pone.0251700)
Supplement: S2 Table — (DOCX) [file pone.0251700.s003.docx]

S2 Table. Change in appetitive sensations, food craving, and impulsiveness in adults consuming isocaloric beverages and solids for 28 days

Beverage Solid

Baseline Day 28 Baseline Day 28 Delta 95% CI

Retrospective Visual Analogue Scale (RVAS)

Hunger 53.3 ± 16.2 41.4 ± 27.8 53.8 ± 15.5 54.7 ± 23.6 -12.8 ± 18.4 (-30.1, 4.5)

Fullness 66.1 ± 15.1 72.2 ± 8.4 66 ± 13.8 67.8 ± 18.6 4.3 ± 20.9 (-15.3, 23.9)

Desire to Eat 68 ± 17.3 73.9 ± 12.1 68.8 ± 16.7 80.1 ± 15.8 -5.4 ± 18.1 (-22.4, 11.6)

How Much 64.9 ± 15.6 69.6 ± 16.7 64.2 ± 17.9 71.9 ± 12.5 -3.0 ± 15.2 (-17.3, 11.3)

Food

Satisfied 78.6 ± 15.6 81.6 ± 11.1 72.3 ± 25.9 76.3 ± 23.8 -1.0 ± 25.2 (-24.7, 22.7)

Food Craving Inventory (FCI)

High Fats 2.1 ± 0.5 2.6 ± 0.5 1.9 ± 0.5 2.7 ± 0.6 -0.2 ± 0.3 (-0.5, 0.1)

Sweets 2.7 ± 0.5 2.8 ± 0.6 2.5 ± 0.7 2.5 ± 0.7 0.1 ± 0.4 (-0.3, 0.5)

Carbohydrates 2.3 ± 0.8 2.6 ±0.3 2.1 ± 0.6 2.4 ± 0.4 0.1 ± 0.3 (-0.1, 0.4)

Fast Food 2.8 ± 0.8 3.0 ± 0.4 2.5 ± 1.0 3.0 ± 0.6 0.0 ± 0.5 (-0.5, 0.5)

Fats

Fruits/ 2.7 ± 0.9 2.9 ± 0.6 2.8 ± 0.8 2.4 ± 0.6 0.5 ± 0 .4 (0.2, 0.9)

Vegetables

Total Score 2.5 ± 0.5 2.8 ± 0.3 2.3 ± 0.5 2.6 ± 0.4 0.1 ± 0.2 (-0.1, 0.3)

Barratt Impulsiveness Scale (BIS)

Attention 9.0 ± 2.4 8.3 ± 1.7 8.9 ± 1.8 9.0 ± 2.3 -1.0 ± 1.4 (-2.4, 0.5)

Cognitive 5.6 ± 1.9 5.5 ± 1.5 5.5 ± 2.6 5.6 ± 1.9 0.3 ± 1.6 (-1.3, 2.0)

Instability

Attentional (2^nd^) 14.6 ± 3.9 13.8 ± 3.0 14.4 ± 3.6 14.6 ± 2.8 -0.6 ± 2.2 (-2.9, 1.7)

Motor 13.3 ± 2.4 14.8 ± 1.75 12.7 ± 2.3 16.3 ± 2.1 -2.0 ± 2.2 (-4.4, 0.3)

Persever- 6.0 ± 1.5 6.4 ± 1.9 5.4 ± 1.6 6.4 ± 1.6 -0.2 ± 1.4 (-1.6, 1.3)

ance

Motor (2^nd^) 19.3 ± 2.6 21.1 ± 3.2 18.1 ± 2.7 22.6 ± 2.1 -2.2 ± 2.6 (-4.9, 0.5)

Self-Control 9.5 ± 2.1 11.5 ± 3.6 9.0 ± 2.2 10.8 ± 3.1 -0.1 ± 2.0 (-2.1, 2.0)

Cognitive 10.3 ± 1.8 11.0 ± 1.2 10.5 ± 2.2 10.4 ± 1.2 1.1 ± 1.1 (0.0, 2.2)

Complexity

Nonplanning (2^nd^) 19.8 ± 1.9 22.5 ± 4.3 19.5 ± 3.7 21.1 ± 3.8 1.0 ± 2.2 (-1.3, 3.3)

Total Score 68.3 ± 5.9 66.9 ± 3.8 67.8 ± 6.1 69.4 ± 4.8 -1.8 ± 4.8 (-6.8, 3.2)

Mean ± SD based on linear model results. The Delta is the (Day 28 – Baseline) Beverage – (Day 28 – Baseline) Solid.
