# Supplementary material for: The effects of the form of sugar (solid vs. beverage) on body weight and fMRI activation: A randomized controlled pilot study
Source: PLoS One. 2021 May 17;16(5):e0251700. doi: 10.1371/journal.pone.0251700 (PMC8128228; doi:10.1371/journal.pone.0251700)
Supplement: S3 Table — (DOCX) [file pone.0251700.s004.docx]

S3 Table. Change in fMRI activation in adults consuming isocaloric beverages and solids for 28 days

Beverage Solid

Baseline Day 28 Baseline Day 28 Delta 95% CI

Reward

Amygdala (R) 0.29 ± 0.86 0.07 ± 0.36 0.03 ± 0.38 0.04 ± 0.23 -0.29 ± 0.83 (-1.23, 0.65)

(L) 0.01 ± 0.33 -0.004 ± 0.24 0.06 ± 0.34 0.09 ± 0.27 -0.07 ± 0.3 (-0.42, 0.27)

Caudate (R) 0.2 ± 0.64 -0.07 ± 0.34 -0.2 ± 0.58 0.03 ± 0.35 -0.6 ± 0.86 (-1.57, 0.38)

(L) -0.07 ± 0.77 0.03 ± 0.3 -0.21 ± 1.02 0.15 ± 0.1 -0.32 ± 1.31 (-1.81, 1.16)

Cingulate Gyrus (R) 0.16 ± 0.33 -0.09 ± 0.16 -0.04 ± 0.51 0.06 ± 0.19 -0.37 ± 0.61 (-1.06, 0.33)

(L) 0.41 ± 0.80 0.03 ± 0.33 -0.05 ± 0.64 0.15 ± 0.21 -0.70 ± 1.06 (-1.9, 0.51)

Dorsal Striatum (R) 0.21 ± 0.50 -0.07 ± 0.26 0.23 ± 0.63 0.18 ± 0.29 -0.13 ± 0.87 (-1.13, 0.86)

(L) 0.09 ± 0.3 -0.11 ± 0.11 0.08 ± 0.56 0.11 ± 0.11 -0.19 ± 0.67 (-0.96, 0.57)

Inferior OFC (R) 0.21 ± 0.76 0.0007 ± 0.44 0.12 ± 0.7 -0.09 ± 0.19 -0.04 ± 0.40 (-0.5, 0.42)

(L) -0.03 ± 0.28 0.09 ± 0.40 0.21 ± 0.62 0.40 ± 0.30 -0.16 ± 0.44 (-0.66, 0.34)

Insula (R) 0.08 ± 0.38 0.44 ± 0.55 0.27 ± 0.57 -0.01 ± 0.45 0.83 ± 0.7 (0.03, 1.62)

(L) -0.08 ± 0.48 0.09 ± 0.24 -0.03 ± 0.62 0.14 ± 0.35 0.09 ± 0.78 (-0.8, 0.97)

Lateral OFC (R) 0.15 ± 1.0 -0.10 ± 0.43 -0.08 ± 0.84 0.23 ± 0.54 -0.81 ± 1.44 (-2.45, 0.83)

(L) -0.05 ± 0.52 0.06 ± 0.36 0.02 ± 0.82 0.26 ± 0.23 -0.20 ± 0.82 (-1.13, 0.73)

Medial OFC (R) 0.58 ± 1.01 -0.04 ± 0.93 -0.13 ± 1.19 0.45 ± 0.73 -1.60 ± 1.59 (-3.41, 0.22)

(L) 0.39 ± 1.0 -0.05 ± 0.44 0.14 ± 0.57 0.32 ± 0.39 -0.72 ± 1.05 (-1.92, 0.48)

PFC (R) 0.22 ± 0.48 -0.06 ± 0.23 0.01 ± 0.34 0.07 ± 0.2 -0.32 ± 0.62 (-1.03, 0.38)

(L) 0.44 ± 1.32 -0.07 ± 0.76 -0.02 ± 0.93 0.02 ± 0.4 -0.65 ± 1.81 (-2.71, 1.42)

Putamen (R) 0.15 ± 0.34 -0.05 ± 0.25 -0.08 ± 0.4 0.12 ± 0.41 -0.28 ± 0.63 (-1.0, 0.43)

(L) 0.04 ± 0.28 -0.03 ± 0.35 0.09 ± 0.52 -0.05 ± 0.2 0.15 ± 0.65 (-0.59, 0.9)

Substantia Nigra (R) -0.07 ± 0.27 0.01 ± 0.3 0.17 ± 0.29 -0.11 ± 0.31 0.39 ± 0.31 (0.04, 0.74)

(L) 0.24 ± 0.58 -0.13 ± 0.44 0.23 ± 0.36 0.12 ± 0.28 -0.25 ± 0.64 (-0.98, 0.47)

Ventral Striatum (R) 0.10 ± 0.39 -0.14 ± 0.16 0.07 ± 0.64 0.13 ± 0.16 -0.29 ± 0.78 (-1.18, 0.60)

(L) 0.27 ± 0.51 -0.14 ± 0.34 0.09 ± 0.72 -0.07 ± 0.21 -0.12 ± 0.91 (-1.16, 0.91)

Energy Homeostasis

Hypothalamus (R) -0.07±0.55 -0.16±0.2 -0.1±0.32 0.19±0.22 -0.4±0.43 (-0.89, 0.08)

(L) 0.02±0.71 -0.06±0.29 -0.04±0.39 -0.004±0.33 -0.06±0.89 (-1.08, 0.95)

Mean ± SD based on linear model results. The Delta is the (Day 28 – Baseline) Beverage – (Day 28 – Baseline) Solid.
