# Supplementary material for: The effects of the form of sugar (solid vs. beverage) on body weight and fMRI activation: A randomized controlled pilot study
Source: PLoS One. 2021 May 17;16(5):e0251700. doi: 10.1371/journal.pone.0251700 (PMC8128228; doi:10.1371/journal.pone.0251700)
Supplement: S1 Protocol — (PDF) [file pone.0251700.s005.pdf]

Protocol Title: The Diet composition and Energy Balance Pilot Study (The DEB Pilot Study)

PI: John W. Apolzan, PhD

Co-PI: Corby K. Martin, PhD

Sub-Investigators:

Owen T. Carmichael, PhD

Nicole N. Fearnbach, PhD

Kishore M. Gadde, MD

Background:

Food form refers to the physical state of foods, i.e., if food is in beverage (liquid) or solid form. Adults do not fully compensate for calories consumed in beverages over the short-term in preload studies; therefore, it is thought that food form can affect energy intake and body weight[1,2]. A meta-analysis of pre-load studies by Almron-Roig suggested that food form affects energy intake[3]. However, previously it has been shown that energy compensation typically takes 2-6 days in humans; thus, the validity of preload studies has been questioned[4,5]. Over four weeks, there is a weight gain with carbohydrate beverage intake but no difference between carbohydrate beverage and solid food form treatments[2]. In another four-week study, participants who consumed sugar-sweetened beverages (SSBs) gained far less weight than expected should no compensation have occurred[6] and in a commentary on the article it was noted that further research is needed to determine if people compensate differently over the long-term to energy consumed in solid vs. liquid form[7]. Similarly, others have called for additional research on the extent to which compensation occurs over the long-term in response to energy from SSBs[8].

The effect of sugar intake on body weight over the long-term is not well understood when a liquid SSB and an SSB consumed in solid form are provided that are similar in macro- and micronutrients. Thus, the purpose of the current study is to test if weight change is differentially affected by consumption of foods that are identical in carbohydrate (i.e. sugar) but vary in food form over 1 month. There will be no instructions provided for any other dietary modifications.

The provided foods that will be consumed by participants daily for 1 month in this study are:

<u>1)</u> 1 20 oz. carbonated sugar sweetened beverage (SSB; 240 kcals). Added ingredients include: ~2.1g whey protein powder.

Or

<u>2)</u> 1 solid food that is made with the SSB syrup concentrate and is equivalent to one 20 oz. soft drink (solid; 240 kcals). Added ingredients: ~30 g water, ~3.0 g gelatin, ~3.5 mg sodium (added using NaCl), ~11.1 mg potassium (added using KCl) and ~11.5 mg calcium (added using CaCl).

Specific Aims:

Aim 1. Test the feasibility of having participants consume liquid and solid foods for 1 month and demonstrate our ability to acquire and analyze fMRI brain activation pre and post dietary intervention with a visual reward paradigm.

Aim 2. Test for change in body weight.

Hypothesis: The SSB group will have no difference in change in body weight compared to the solid group (between group effects). The SSB and solid groups will have increased weight compared to baseline (within group effects).

Changes in brain activation will be measured and tested for differences among the groups. It is hypothesized that change in brain activity will not differ between the SSB and solid groups. But, it is hypothesized that both the SSB and solid group will experience change in activation in reward-related areas of the brain compared to baseline.

The foods listed above will be provided to participants with instructions to consume one serving per day. The energy content of food provision in the SSB and solid groups will be 240 kcal/day (SSB and solid groups will be isocaloric). Participants will receive no instructions for other dietary modifications; i.e., they will not be told to modify what or how much other food they consume.

Inclusion and Exclusion Criteria:

The target study group will be 20 men and women aged 21 to 65 years. 10 participants will be randomized to the SSB and solid groups, respectively. The participant population will include normal weight, overweight and obese (BMI 20.0 to 40.0 kg/m^2) individuals. All inclusion/exclusion criteria will be based upon self-reported medical history/checklist. Participants must be able to consume study foods and be willing to archive their brain scans and eye tracking data. Participants who consume SSBs will be asked to maintain their regular SSB consumption in addition to the SSB (or solid food) associated with their random assignment). Participants will be stratified based on sex and BMI class.

Exclusion Criteria:
Health Conditions:

- Diabetes (Type 1 or 2)
- Prediabetes
- Active cancer
- Serious digestive disorders
- Other conditions that affect metabolism or body weight
- Uncontrolled Thyroid disorder (controlled = 6 months of medication)
- Unable to consume study foods
- Non- weight stable (more than 10 lbs. weight gain or loss in last 6 months)
- Pregnancy
- Intentions of becoming pregnant in the next 2 months
- Women who have undergone partial hysterectomy with intact ovary function
- Any uncorrected vision problems
- Color-blindness
- Left-handed
- Current or past alcohol or drug abuse problem

- Have internal metal medical devices including cardiac pacemakers, aortic or cerebral aneurysm clips, artificial heart valves, ferromagnetic implants, shrapnel, wire sutures, joint replacements, bone or joint pins/rods/screws/clips, metal plates, metal fragments in your eye, or non-removable metal jewelry such as rings.
- Other contraindications to magnetic resonance scanning
- Unable or unwilling to complete the imaging procedures for the duration of the MRI scan due to claustrophobia or other reason.
- Allergy or intolerance to study product ingredients.
- Any other medical, psychiatric or behavioral factors that in the judgment of the Principle Investigator that may interfere with study participation or the ability to follow the protocol

Medications/Non-Drug Therapies (within ~4 weeks prior to Screening):

- Weight loss medications, diet pills
- Non-topical steroids
- Other medications that may affect fluid balance or weight

Lifestyle:

- Consume more than seven 12 oz. sugar-sweetened beverages per week (~ 1, 12 oz. SSB/day; diet soft drinks are ok)
- Consume > 3 drinks/day of any alcoholic beverage
- Smoker. (Former smokers must be smoke free for 6 months)
- You plan to move out of the study area within the next 3 months, or plan to be out of the study area for more than 1 week (~7 days) during the course of the study.

Number of Subjects
We will enroll 20 right handed men and women aged 21-65 y with a BMI of 20.0-40.0 kg/m^2.

Recruitment Methods
Traditional PBRC recruiting methods will be utilized by marketing and the recruiting core.

The Advertising and Recruitment Cores are responsible for recruiting potential participants for clinical trials from a population that includes a wide range of people of various ages, ethnic backgrounds and with varying degrees of health. Pennington Biomedical has a team of full-time employees dedicated to the recruitment and initial screening of clinical trials. Potential subjects will be identified via a large database of previous study participants along with the use of web, print, and media advertising. The Recruitment and Advertising Cores create and implement individualized, trial-specific advertising and awareness campaigns, including mass media, traditional advertising, and novel methods including social media, digital and email marketing. All advertising and awareness are approved by our on-site IRB to ensure ethical and disclosure standards are met. The PBRC database will also be utilized to find potential subjects for this study.

Study Timelines
Participants will come in for a screening visit. Then approximately a week (~ 7 days) later, participants will come in their Week 0 (baseline) study visit and be provided with ~1 week (~7

days) of food provision. This visit will occur based on self-reported luteal phase of menstrual cycle in women. Next, at approximately 1 week (~day 7), participants will return to the center for an adherence visit and further provision of study foods (Week 1). At approximately 2 weeks (~day 14), participants will return to the center for an adherence visit and further provision of study foods (Week 2). At approximately 3 weeks (~day 21), participants will return to the center for an adherence visit and further provision of study foods (Week 3). Finally, participants will come for their Week 4 assessment and adherence visit. Thus, participation will last approximately 5-6 weeks in total.

Study Endpoints
Study Design: A randomized parallel arm design trial examining solid sugar vs. SSB treatments will be performed. The primary study endpoints are feasibility and change in body weight.

Procedures Involved
Overview:
Participants will arrive at the center following a minimum 10 hour fast. They will undergo anthropometric measurements, psychological and behavioral questionnaires, and fMRI.

Table 1. Schedule of Procedures

		Intervention Period				
**Visit Assessments**	**Screening Visit**	**Week 0**	**Week 1**	**Week 2**	**Week 3**	**Week 4**
Informed Consent	X					
Anthropometrics (height, body weight)	X					
Anthropometrics (body weight, waist and hip circumference, and bioelectrical impedance)		X				X
Brief Lifestyle Interview and non-quantified food record	X					
Contact and Demographic Information	X					
Food Taste Test	X					
Food Craving Inventory (FCI), Yale Food Addiction Survey (YFAS) , Liking and Wanting Procedure, Retrospective Visual Analogue Scales (RVAS) , Barratt Impulsiveness Scale, Delayed Discounting Task, and Food Familiarity and Liking Procedure		X				X
Urine Pregnancy Test		X				X
fMRI (fasting and fed state)		X				X
Randomization		X				
Study Product Dispensed		X	X	X	X	
Adherence check/calculations (self-report and container return), provision of study foods			X	X	X	X

Dose: The energy content of food provision in the SSB and solid groups will be 240 kcal/day. Participants are to consume 1 unit per day of the study.

Diet: Participants will receive no instructions for other dietary modifications; i.e., they will not be told to modify what or how much other food they consume.

Food Acceptability Form: Participants will be asked to rate the study foods for appearance, odor, flavor, texture, sweetness, and overall acceptability at screening. Each participant will be asked to circle the number from 1-9 for each of the characteristics after consuming the study foods.

Psychological Assessments:

Food Craving Inventory (FCI). The FCI[9,10] is a 28-item measure of specific food craving. The FCI assesses the frequency with which an individual experiences a craving for a particular food. The measure consists of 4 empirically-derived factors: High fats, Sweets, Carbohydrates/starches, and Fast food fats. All items are scored in a 1 (Never) to 5 (Always) multiple choice format. The measure has been shown to be reliable and support has been found all aspects of validity.

Yale Food Addiction Scale (YFAS). The YFAS[11] is a 27-item self-report questionnaire used to identify individuals showing tendencies for addictive-like behaviors towards certain types of foods, such as those high in fat or sugar. The majority of the items are rated on a 5 point Likert scale, with responses ranging from "Never" to "Four or more times per week or daily" or a two point Likert scale consisting of "yes" or "no" responses. The measure also allows subjects to subjectively identify specific problem foods. Validity for the YFAS has been established.

Liking and Wanting Procedure. The liking and wanting procedure assesses explicit liking and wanting, followed by implicit wanting for the same target food stimuli[12,13]. The explicit task quantifies hedonic response, measured with visual analogue scales (VAS), to 16 food stimuli used in the implicit wanting task. A forced choice method is used to measure implicit wanting by presenting paired food stimuli from four food categories and asking participants to select the food they "most want to eat now". Frequency of selections for each food category quantifies relative preference and reaction time measures implicit behavior change.

Retrospective Visual Analogue Scale (RVAS). RVAS will be used to measure average ratings of appetitive sensations that participants experienced over the past week. This method of collecting VAS data has been found to be consistent with daily assessments of satiety[14], and has been found to be reliable and valid measure of subjective states of appetite related to energy intake[15].

Barratt Impulsiveness Scale (BIS). It is a 30 item scale that yields six first order factors (attention, motor, self-control, cognitive complexity, perseverance, and cognitive instability impulsiveness) and three second order factors (attentional, motor, and non-planning impulsiveness). It is a self-report measure of impulsive personality traits.

Delay Discounting Task. Immediate rewards are preferred to delayed rewards of higher value and the amount of this "delayed discounting" provides an index of impulsivity. Weight loss outcomes could be influenced by discounting the long-term rewards of weight loss in relation to immediately available food rewards [16]. Images of a preferred food will be presented and participants will be asked "Would you rather eat this food now or receive $10 tomorrow?" (Participants select the "food" or "money" responses). Reaction time will be recorded and the food image will be used with different monetary choices ($1, $5, $10, $20, $50, $100) with delay times of now, 4 hours, tomorrow, 1 week, 2 weeks, and 1 month.

Food Familiarity and Liking Procedure. The Food Familiarity and Liking Procedure asks participants to evaluate a total of 105 images of food and control photos shown in the fMRI scanner paradigm following completion of all scanning procedures. Participants will see each photo and be asked whether they know what the food or item is and rate how much they like the food or item on a visual analog scale.

Here is the schedule for the screening and clinic visits:

Screening Visit: About 1.5 hours –Fasting (nothing to eat or drink other than water for at least 10 hours prior to visit)

This visit will take place in the Outpatient Clinic Department of Pennington Biomedical. The testing this day includes the following procedures:

- Informed Consent
- Current medication and supplement use
- Demographic and Lifestyle Questionnaire (including contact information)
- Lifestyle Interview
  - To discuss your understanding of the study and for the study staff to know what kinds of barriers may make it difficult for you to participate in the study. Such lifestyle barriers include your work or school schedule, outside activities, and other responsibilities may prohibit your from enrolling.
- Height
- Weight
- Food Taste Test

Week 0: About 4 hours – Fasting (nothing to eat or drink other than water for at least 10 hours prior to visit) after at least 7 hours of rest the previous evening

This visit will take place in the Outpatient Clinic Department of Pennington Biomedical. The testing this day includes the following procedures:

- Anthropometric Measurements – (body weight, waist and hip circumference, and bioelectrical impedance)
- Psychological and Behavioral Questionnaires
- Females of child-bearing potential will have a urine pregnancy test
- MRI/fMRI – fasting and fed state
- Treatment consumption – fed state fMRI
- 1 week supply of study product dispensed

Week 1: About 30 minutes – Non-Fasting

- Adherence check
- 1 week supply of study product dispensed

Week 2: About 30 minutes – Non-Fasting

- Adherence check
- 1 week supply of study product dispensed

Week 3: About 30 minutes – Non-Fasting

- Adherence check
- 1 week supply of study product dispensed

Week 4: About 4 hours – Fasting (nothing to eat or drink other than water for at least 10 hours prior to visit) after at least 7 hours of rest the previous evening

This visit will take place in the Outpatient Clinic Department of Pennington Biomedical. The testing this day includes the following procedures:

- Anthropometric Measurements – (body weight, waist and hip circumference, and bioelectrical impedance)
- Psychological and Behavioral Questionnaires
- Females of child-bearing potential will have a urine pregnancy test
- MRI/fMRI – fasting and fed state
- Treatment consumption – fed state fMRI
- Adherence check

All participants need to come in within ± 2 days of their scheduled visit date.

Phone calls, text messages and email are also permissible by the adherence coach as adherence checks during the dietary intervention. Also adherence coaches can request remote motoring i.e. pictures of study foods.

<u>Study Procedures:</u>

As previously stated, the foods listed above will be provided to participants with instructions to consume one serving per day. If they happen to skip a day, participants will be instructed to consume the treatment on the following day on top of their normal daily provision. The energy content of food provision in the SSB and solid groups will be 240 kcal/day (SSB and solid groups will be isocaloric). <u>Participants will receive no instructions for other dietary modifications; i.e., they will not be told to modify what or how much other food they consume.</u>

fMRI: Reward Activation

At baseline and follow-up, subjects will arrive to PBRC after an overnight fast where they will participate in a fMRI scanning protocol to assess brain response patterns to reaction times and attentional processing to food and control images. The scanning session will begin with a structural scan where the participant will be asked to lie as still as possible while we scan their brain. The paradigm will immediately follow in which subjects will view images of pictures with up to seven categories: 1) High Sugar/High Fat (HS/HF), 2) High Complex Carbohydrate/High Fat (HCC/HF), 3) Low Carbohydrate/High Protein/High Fat (LC/HP/HF), 4) High Sugar/Low Fat (HS/LF), 5) High Complex Carbohydrate/Low Fat (HCC/LF), 6) Low

Carbohydrate/High Protein/ Low Fat (LC/HP/LF), and/or 7) Control Images presented in an event design. Subjects will also be asked to solve a mental task which may include clicking buttons to rate pictures. Additionally, we may collect eye tracking data during the fMRI scanning protocol to further assess attentional processing of food-related visual cues.

Fasting vs. Fed Neural Activation Patterns:

Subjects will complete all scanning procedures in a fasting state and additionally post meal consumption. After completing the fMRI scan in the fasted state, subjects will participate in a meal challenge by consuming their dietary treatment and after will undergo the same fMRI image protocol as before to assess changes in activation in response to fasted and fed conditions. Recently Smeets et al. investigated how neural activation patterns in response to caloric and non-caloric stimuli are modulated by hunger (fasted) and satiety (fed). They showed a significant interaction between hunger and energy content in the solutions and that tasting energy during satiety resulted in greater activation patterns than during hunger[17]. For these reasons, we will assess reward activation patterns in response to food cues in the fasted and fed state.

MR scan preparation

At the phone screen, each participant will be asked a series of questions regarding possible contraindications to MR scanning. Study personnel will ask additional questions and clarify any uncertainties with the participant on possible contraindications to MR scanning at the baseline visit. On the day of MR scanning, the MR technician will rescreen the participant out of caution. Participants scheduled for a functional MRI scan that involves viewing images, listening to sounds, and/or pressing buttons to respond to questions may be asked to do a practice session in front of a computer (outside the scanner) on the day of the scan. During the practice session, the participant will view similar images, hear similar sounds, and press computer keys in similar ways to familiarize them to what will happen inside the scanner. The MR technician will then ensure that all metallic items are removed from the exterior of the body of the participant, and that clothing that might cause image artifacts are removed. For brain scans, some participants wearing simple cotton clothing are expected to be able to remain clothed. The MR technician will provide the participant with earplugs to reduce scanner noise. The MR technician will then place the participant on the MR table, supplying cushioning under the neck and legs as needed to ensure participant comfort. A blanket will be provided to participants that feel cold on the MR table. The MR coil will then be placed over the head and the participant is then inserted into the MRI tube by the MR technician.

Physiological monitoring and stimulus devices

Physiological monitoring will be applied to the participant. The reason for physiological monitoring is that it provides ancillary data that improves the quality of the collected MRI data. Physiological stimulation devices will be applied to measure the response of the brain to stimuli, which is of scientific interest. The devices are listed below.

Respiratory monitor: The respiratory monitor is a belt that fits around the chest at the level of the diaphragm of the participant. The MRI machine records the rise and fall of the diaphragm during scan acquisition. Dynamic MRI data, especially functional MRI and perfusion MRI of the brain, is commonly corrected post hoc by regressing this respiratory signal from the collected

time series data. The belt is made of comfortable material and stretches amply so it does not restrict the ability to breathe normally.

Pulse oximeter: The pulse oximeter is an optical sensor that clips onto one of the fingers of either hand or toes of either foot. It shines a light through the skin to gather proxy measures of blood oxygenation while the scan is being collected. Dynamic MRI data, especially functional and perfusion MRI, is commonly corrected by regressing the time course of peripheral blood oxygenation out of the raw time series data acquired. The sensor attaches snugly to the finger but generally does not pinch or cut off the flow of blood.

Image and video display screen: Participants view images projected from a screen through a mirror mounted on the MRI head coil. Commonly deployed experimental paradigms display images during fMRI data collection to assess responses of the brain to various visual stimuli.

Headphones: MR-safe headphones are worn inside the head coil and over the ears.

Button boxes: In functional MRI experiments, participants are commonly asked to indicate responses to questions about the images, sounds, smells, and tastes exposed to them in the scanner by pressing buttons on button boxes placed in their hands.

MR scanning

The scanner technician runs a series of sequence acquisitions, each of which quantify a different aspect of tissue structure and function, or covers a slightly different portion of the anatomy. Each sequence acquisition emits a series of buzzing or grinding noises over the course of 1 to 10 minutes while data is acquired. Before each sequence acquisition, the technician communicates with the participant via a speaker and microphone inside the scanner bore; the participant is asked about his/her comfort and is reminded to lie perfectly still during the subsequent sequence acquisition. The technician has an unobstructed view of the participant to identify movement during acquisition or other signs of discomfort. After scanning, the participant is removed from the scanner tube and assisted out of the scanner suite.

Statistical analysis. The study will use an intent to treat approach. Following evaluation of data normality, a mixed model ANOVA will be used to test if change on the outcome variables differed between treatment and control across time. LS Means will also test if change from baseline was significantly different from 0. Fixed effects will include age and sex.

Power Analysis:

Since this is a pilot and feasibility study, power calculations have not been performed. This study will allow us to obtain crucial feasibility data as well as data to perform future power calculations for a larger grant.

Data Banking

The PIs and co-investigators will have access to the data for future analysis and they may be shared with outside investigators.

Data Management

The data management will be conducted by the Pennington Biomedical Research Computing Group. There will be limited access to all data including locked cabinets for paper files and password protected computers for electronic data. Identifiers will be kept separate from the data.

Provisions to Monitor the Data to Ensure the Safety of Subjects
The Principal Investigators (PIs) are responsible for monitoring the data, assuring protocol compliance and conducting safety reviews at the specified frequency [quarterly]. During the review process, the Principal Investigators will evaluate whether the study should continue unchanged, requires modification/amendment or should close to enrollment.

The Principal Investigators or the Institutional Review Board (IRB) or have the authority to stop or suspend the study or require modifications.

This protocol presents minimal risks to subjects, and adverse events or other problems are not anticipated. In the unlikely event that such events occur, the PIs are responsible for reporting to the IRB and any appropriate funding and regulatory agencies any serious, unanticipated and related adverse events or unanticipated problems involving risks to subjects or others. The investigators will apprise fellow investigators and study personnel of all adverse events that occur during the conduct of this research project. The investigator will meet this obligation through email as they are reviewed by the Principal Investigators. The study's Research Monitor(s) and decision-making bodies will be informed of serious adverse events within 10 working days of the event becoming known to the Principal Investigators. If there are unanticipated problems involving risks to subjects or others, these should be reported to the IRB per HRPP Policy 8.0.

The sub-investigator, Kishore M. Gadde, MD, will review adverse events.

Withdrawal of Subjects
Subjects may be withdrawn from the study if he/she misses study visits or food provision and will be notified of their withdrawal via telephone or mail.

If a subject voluntarily withdraws from the study, no additional data will be collected and they will be considered drop outs in the study.

Risks to Subjects
This study does not involve major risk to study participants.

- **Fasting for 10 hours:** There is a possibility that fasting for 10 hours may make you feel nauseous.
- **Self-reported Questionnaires:** There are no anticipated risks from completing self-report questionnaires. Due to the sensitive nature of the questionnaires, you may skip any questions that you do not wish to answer.
- **Bioelectrical Impedance Analysis (BIA) Measurement:** You will be asked to change into a gown and to remove all footwear and socks/stockings. Once changed and barefoot, you will be asked to stand on a scale (similar to a large gym scale), and you may be asked to hold on to hand electrodes on each side of the scale. You will be asked to step off of the scale once the measurement is complete (less than one minute).

- There is no risk associated with the BIA measurement. **However, subjects with medical implants such as a pacemaker or metal joint replacements cannot be measured on the machine.**

- **Weight Gain:** There is a possible chance that the Calories will slightly increase body weight.
- **Brain MRI:** This scan is performed to measure the size of your brain and lasts approximately ten minutes. You will change into a hospital gown and remove all objects containing metal from your body. During the scan, you will lie on your back on the scanner table with your head in a cradle. The scanner table will then move you into the magnet. During the scan, you will hear loud tapping noises. You will be given head phones for protection from the scanner noise and can listen to music during the scan if desired. You will also be given a call button should you need the MRI tech during the exam. This scan is for research purposes only and not for diagnostic treatment.
    - **MRI Risks:**

There are no known biological risks associated with magnetic resonance scanning. It has been used routinely for over 20 years. It produces side effects in very few situations. Those situations include:

- **Metal:** Because the magnetic resonance machine uses a magnetic field, it can move any metallic objects that are inside the body. This disruption of metal inside the body is extremely dangerous to you and may even be life threatening. If you think you may have a cardiac stent, metallic implant, metallic piercings, shrapnel, or any other metallic material in your body, it is of utmost importance that you alert the study coordinator or MR technician. If you have metallic materials in your body that cannot be removed, we will exclude you from this study for your safety.
- **Electronics:** Magnetic resonance imaging involves the use of radio frequency energy that can disrupt the functioning of electronic devices. If you think you might possess a pacemaker or any other electronic medical device inside your body, it is of utmost importance that you inform the study coordinator or MR technician. If you have any such electronic devices we will exclude you from this study for your safety.
- **Tattoos and cosmetics:** Some tattoos and cosmetics contain metallic materials that can heat up during scanning, especially if they are located on the part of the body being scanned. If the metallic material heats up enough, you may feel an uncomfortable burning sensation, and a skin burn may develop. If you have any tattoos or cosmetics that might contain metallic materials, please alert the study coordinator or MR technician. If you feel a burning sensation on your skin, alert the study coordinator or MR technician. In some cases, the amount of metallic material in the area being scanned is so excessive that the scan must be stopped. In other cases, a cold compress placed over the metallic material will be used to prevent the burning sensation.
- **Confinement:** During the MR scan, you will be lying down on a table inside of a metal tube. The metal tube is a confined place. This might produce a feeling of claustrophobia, which can be distressing. If you have experienced claustrophobia in the past, you might become too distressed to complete the scan. If you become distressed during the scan due to confinement in the scanner tube, please alert the MR technician and the scan will be halted.

- **Noise:** The MRI machine creates a loud, rhythmic noise that sounds like grinding or churning. This can be distressing to those who are sensitive to loud noises. You will be provided with earplugs to reduce the noise. But if you find the machine noises distressing, alert the MR technician and the scan can be halted.
- **Peripheral nerve stimulation:** During the MR scan, the magnetic field around your body goes through rapid changes. These changes are all within safety limits set by the Food and Drug Administration. But, some people experience twitching in the nerves of their arms or legs as a result of these magnetic field changes. This twitching is generally not painful, and it stops at the end of the MR scan. But the feeling of inadvertent muscle twitching may make you feel disoriented or uncomfortable. If you experience this and wish to stop the scan as a result, please tell the MR technician.
- **Physical frailty:** The MR technologist performing the scan has received extensive training in how to position all participants, including elderly ones, in the MRI machine safely and comfortably. However, some older people have a more difficult time walking or moving their bodies due to arthritis and other conditions. There is a slight chance that these individuals could feel discomfort or fall during transitions into or out of the MRI scanner. The technologist will ensure that the walkway to the scanner is safe for you to walk on, will place cushioning on the scanner table for your comfort, and will carefully guide your movements around the scanner to minimize this risk.
- **Venous thromboembolism:** In some elderly or obese individuals, lying down perfectly still for multiple hours can slightly increase the risk that blood clots develop in the blood vessels. These blood clots can be hazardous to your health. The technologist will make every effort to keep your time in the MRI machine as short as possible to reduce this risk. Also, you will have breaks during your time in the MRI machine, and during these breaks the technologist will ask you to move your arms and legs and reposition your body to get comfortable. Moving around in this way reduces your risk of blood clots.

Incidental Findings

We will follow the recommendations of a Presidential panel in handling incidental findings on MRI. First, we indicate to the participant whether or not there is a well-established set of incidental findings for the scan they are undertaking, and if so what those incidental findings are. For scans without a well-established set of incidental findings, we will provide a list of incidental findings that we feel may be possible. We will then describe the difference between clinically actionable incidental findings and non-clinically-actionable incidental findings. We will then ask the participant to decide whether they want to be informed of non-clinically-actionable incidental findings. Participants will be informed that informing them of clinically-actionable incidental findings is required for participation in the study. In the event that study personnel identify MR abnormalities, they will consult with a radiologist who will determine the clinical relevance of the abnormalities. Participant identity will not be shared with the radiologist in this event. If the radiologist determines that an MR abnormality is relevant to personal health, the radiologist will then determine whether the finding is clinically actionable. In the event that the finding is clinically actionable, or if the participant consented to be informed of non-clinically actionable incidental findings, study personnel will inform the participant and will suggest discussing the findings with the personal health professional of the participant. In the event of an incidental

finding that is to be released to the participant, the imaging findings flow from the study staff, to a radiologist, to the Investigator who explains the findings with the participant.

Potential Benefits to Subjects

There are no direct benefits for subjects for participation in the study. The MR scan data collected for the study is not designed to be used by a doctor to evaluate physical health the way a scan done in a hospital or clinic is. That means that any abnormalities in the body that are relevant to personal health will not necessarily be noticed by study personnel. However, MR scanning may result in discovery of an unexpected incidental finding as described above. Therefore, there is a small chance that this study will provide benefit by revealing problems with personal health that would not have been discovered without MR scanning.

A participant might also benefit by learning new information about the structure and function of the brain. Such information may be interesting to the participant.

Sharing of Results with Subjects

The participants may receive a structural image of their brain if an inquiry is made.

Setting

We will utilize the clinical facilities as well as the neuroimaging center at Pennington Biomedical Research Center to conduct this study.

Compensation

Subjects will receive $75 for completing Week 0 baseline visit, $10 for completing Week 1 visit, $10 for completing Week 2 visit, $10 for completing Week 3 visit, and $125 for completing Week 4 visit, for a maximum of $230.

Number of Subjects

We aim to complete the study on a total of 20 subjects.

Provisions to Protect the Privacy Interests of Subjects

All attempts will be made to maintain a subject's privacy. Safeguards such as password protected computer and networks have been put in place in order to limit access to subject data.

Subjects will be given ample time to read over the consent, ask questions, and agree to participate in the research study. Subjects may decline answering questions they are not comfortable with. Each procedure will be explained to the subject before it is performed.

We will always ensure the privacy of the subjects.

Compensation for Research-Related Injury

No compensation will be provided for research-related injury.

Economic Burden to Subjects

All study-related tests and procedures will be at no cost to the subject. The subject will incur transportation costs in getting to PBRC.

Consent Process

The P.I. or one of the designated clinic staff will obtain informed consent in the clinic during the first visit. Ample opportunity will be given for the subject to review the consent and ask any questions prior to signing the consent form. If subjects wish, they can take the form home and return at a different visit. Also, as we are aware consent is an ongoing process.

Data Sharing

The participant is asked to allow MRI scans to be stored and used for research at a later time. Participants that refuse to have scans kept for future research will be excluded from the study. The MRI scans will be stored indefinitely. The scans may be given to other investigators or MRI technology companies for future research as well. The future research may take place at Pennington Biomedical and may involve Pennington Biomedical Researchers in this study. The MRI scans may be shared with faculty at the LSU School of Kinesiology. The future research may not take place at Pennington Biomedical Research Center and may not be reviewed by Pennington Biomedical Research Center's Institutional Review Board. For privacy and confidentiality, MRI scans will be labeled with a unique series of letters and numbers. Pennington Biomedical will store MRI scans with this unique identifier. The research done with MRI data may help to develop new products in the future, or may be used to establish a diagnostic test that could be patented or licensed. The participant will not receive any financial compensation for any patents, inventions or licenses developed from this research.

References:

1. Mattes RD. Dietary compensation by humans for supplemental energy provided as ethanol or carbohydrate in fluids. *Physiol Behav.* 1996;59(1):179-187.
2. DiMeglio DP, Mattes RD. Liquid versus solid carbohydrate: effects on food intake and body weight. *Int J Obes Relat Metab Disord.* 2000;24(6):794-800.
3. Almiron-Roig E, Palla L, Guest K, Ricchiuti C, Vint N, Jebb SA, Drewnowski A. Factors that determine energy compensation: a systematic review of preload studies. *Nutrition Reviews.* 2013;71(7):458-473.
4. Bray GA, Flatt JP, Volaufova J, Delany JP, Champagne CM. Corrective responses in human food intake identified from an analysis of 7-d food-intake records. *Am J Clin Nutr.* 2008;88(6):1504-1510.
5. Saris WHM. Limits of human endurance: lessons from the Tour de France. . In: Kinney JM, Tucker HN, eds. *Physiology, stress, and malnutrition: functional correlates, nutritional intervention.* . Philadelphia: Lippincott-Raven Publishers; 1997:451-462.
6. Reid M, Hammersley R, Duffy M, Ballantyne C. Effects on obese women of the sugar sucrose added to the diet over 28 d: a quasi-randomised, single-blind, controlled trial. *The British journal of nutrition.* 2013:1-8.
7. Allison DB. Liquid calories, energy compensation and weight: what we know and what we still need to learn. *The British journal of nutrition.* 2013:1-3.
8. Kaiser KA, Shikany JM, Keating KD, Allison DB. Will reducing sugar-sweetened beverage consumption reduce obesity? Evidence supporting conjecture is strong, but evidence when testing effect is weak. *Obes Rev.* 2013;14(8):620-633.
9. White MA, Grilo CM. Psychometric properties of the Food Craving Inventory among obese patients with binge eating disorder. *Eat Behav.* 2005;6(3):239-245.
10. White MA, Whisenhunt BL, Williamson DA, Greenway FL, Netemeyer RG. Development and validation of the food-craving inventory. *Obes Res.* 2002;10(2):107-114.
11. Gearhardt AN, Corbin WR, Brownell KD. Preliminary validation of the Yale Food Addiction Scale. *Appetite.* 2009;52(2):430-436.
12. Finlayson G, King N, Blundell J, E,. Liking vs. wanting food: Importance for human appetite control and weight regulation. *Neuroscience & Biobehavioral Reviews.* 2007;31(7):987-1002.
13. Finlayson G, king N, Blundell J, E,. The role of implicit wanting in relation to explicit liking and wanting for food: Implications for appetite control. *Appetite.* 2008;50(1):120-127.
14. Womble LG, Wadden TA, Chandler JM, Martin AR. Agreement between weekly vs. daily assessment of appetite. *Appetite.* 2003;40(2):131-135.
15. Flint A, Raben A, Blundell JE, Astrup A. Reproducibility, power and validity of visual analogue scales in assessment of appetite sensations in single test meal studies. *Int J Obes Relat Metab Disord.* 2000;24(1):38-48.
16. Appelhans BM. Neurobehavioral Inhibition of Reward-driven Feeding: Implications for Dieting and Obesity. *Obesity.* 2009;17(4):640-647.
17. van Rijn I, de Graaf C, Smeets PA. Tasting calories differentially affects brain activation during hunger and satiety. *Behavioural brain research.* 2015;279:139-147.
